# *Lactobacillus acidophilus* ATCC 4356-derived metabolites inhibit biofilm formation and motility in *Listeria monocytogenes*

**DOI:** 10.1007/s10482-026-02457-w

**Published:** 2026-11-12

**Authors:** Sthefânia Dalva da Cunha Rezende, Isabela Sguilla Rotta, Hugo Felix Perini, Pedro Henrique Oliveira Alves, Marcos Vinicius da Silva, Deyse Christina Vallim, Rodrigo de Castro Lisboa Pereira, Felipe Alves de Almeida, Uelinton Manoel Pinto, Aline Dias Paiva

**Affiliations:** 1 Department of Microbiology, Immunology and Parasitology, Federal University of Triângulo Mineiro, Uberaba, MG Brazil; 2 Department of Education in Science, Mathematics and Technology, Federal University of Triângulo Mineiro, Uberaba, MG Brazil; 3 Bacterial Zoonoses Laboratory, Oswaldo Cruz Institute, FIOCRUZ, Rio de Janeiro, Brazil; 4 Department of Microbiology, Institute of Biotechnology Applied to Agriculture, Federal University of Viçosa, Viçosa, MG Brazil; 5 Laboratory of Food Microbiology, Food Research Center, Department of Food and Experimental Nutrition, São Paulo University, São Paulo, Brazil

**Keywords:** Foodborne pathogen, Lactic acid bacteria, Cell-free supernatant

## Abstract

*Listeria monocytogenes* is a major foodborne pathogen whose persistence in food-processing environments is strongly associated with its ability to survive under a wide range of environmental conditions and form biofilms on different surfaces. Metabolites produced by beneficial lactic acid bacteria may represent a natural source of antimicrobial and antibiofilm compounds capable of affecting bacterial behavior even at subinhibitory concentrations. This study investigated the effects of the cell-free supernatant of the probiotic strain *Lactobacillus acidophilus* ATCC 4356 (*La*-CFS) on growth, biofilm formation, motility, and the expression of selected quorum sensing- and motility-associated genes in *L. monocytogenes*. The antibacterial activity and minimum inhibitory concentration (MIC) of *La*-CFS were determined against multiple *L. monocytogenes* strains. Extracellular signaling activity was evaluated using the AI-2-responsive *Vibrio harveyi* BB170 bioluminescence reporter assay, while the effects of sub-MIC concentrations on biofilm formation and swimming and swarming motility were assessed using phenotypic assays. The expression of *luxS*, *agrC*, *flaA*, and *motA* was analyzed by RT-qPCR. *La*-CFS inhibited the growth of all tested *L. monocytogenes* strains, whereas sub-MIC concentrations induced a transient delay in bacterial growth without significantly affecting the final optical density. Cell-free supernatants from the evaluated *L. monocytogenes* strains induced bioluminescence in the *V. harveyi* BB170 reporter assay, indicating extracellular signaling activity detectable by an AI-2-responsive system. At sub-MIC concentrations, *La*-CFS significantly reduced biofilm formation and impaired swimming and swarming motility in a strain- and concentration-dependent manner. Transcriptional analysis of *L. monocytogenes* CLIST 3833 exposed to *La*-CFS at 0.5 × MIC for 24 h revealed significant downregulation of the motility-associated genes *flaA* and *motA* (p < 0.05), whereas the expression of *luxS* and *agrC* was not significantly altered. These findings demonstrate that metabolites present in the cell-free supernatant of *L. acidophilus* ATCC 4356 can impair biofilm formation and motility in *L. monocytogenes*, supporting further investigation of *La*-CFS as a potential source of bioactive compounds for controlling the persistence of this pathogen in food-processing environments.

## Introduction

*Listeria monocytogenes* is a widespread foodborne pathogen responsible for listeriosis, a severe infection that particularly affects pregnant women, neonates, older adults, and immunocompromised individuals (WOAH 2021). Food contamination by *L. monocytogenes* can occur at multiple stages of the production chain (Meireles et al. 2016). This bacterium is notable for its ability to survive and grow under a broad range of environmental conditions, including refrigeration temperatures (Finn et al. 2023). Its persistence on food-contact surfaces and processing equipment represents a major challenge for food safety, increasing the risk of cross-contamination and complicating sanitation and decontamination procedures (Mazaheri et al. 2021; Ribeiro et al. 2023).

In addition to its ability to tolerate environmental stresses, the persistence of *L. monocytogenes* in food-processing environments can also be attributed to its capacity to establish and maintain biofilms. Biofilm development is a multifactorial process influenced by environmental conditions, bacterial adhesion and motility, and several regulatory networks, including quorum sensing (QS)-related systems (Patel et al. 2025). QS generally involves the production, release, and detection of extracellular signaling molecules known as autoinducers, allowing bacterial populations to coordinate specific collective behaviors and synchronization of target gene expression in response to changes in cell density and environmental conditions (Salman et al. 2023).

Among the QS systems described in *L. monocytogenes*, the accessory gene regulator (Agr) system has been associated with biofilm formation, surface colonization, and virulence-related traits (Riedel et al. 2009; Yu et al. 2022). The *agr* locus comprises the four-gene operon *agrBDCA*. *agrD* encodes the precursor of the autoinducing peptide (AIP), which is processed and exported through the activity of AgrB. The mature AIP interacts with the membrane-associated histidine kinase AgrC, leading to activation of the response regulator AgrA and subsequent modulation of target genes (Rieu et al. 2007; Guerreiro et al. 2022).

The LuxS/AI-2 system, which is widely distributed among bacterial species and has been implicated in interspecies signaling, has also been associated with biofilm formation in *L. monocytogenes* (Challan Belval et al. 2006; Liu et al. 2022; Li et al. 2025). LuxS participates in the activated methyl cycle by catalyzing the conversion of S-ribosylhomocysteine into homocysteine and 4,5-dihydroxy-2,3-pentanedione (DPD), which spontaneously generates molecules collectively referred to as autoinducer-2 (AI-2) (Bassler 1999; Federle and Bassler 2003). However, the contribution of LuxS and AI-2 to *L. monocytogenes* biofilm development appears to be complex, indicating that these phenotypes are controlled by interconnected metabolic and regulatory pathways rather than by a single signaling system.

Lactic acid bacteria (LAB) have attracted considerable interest as a source of bioactive compounds because of their long history of safe use in food production, particularly in fermented foods and as bioprotective cultures. Their metabolic versatility enables the production of a wide range of compounds, including organic acids, bacteriocins and other antimicrobial peptides, hydrogen peroxide, exopolysaccharides, biosurfactants, and diverse low-molecular-weight metabolites (Abedin et al. 2024; Banicod et al. 2026). Beyond direct antimicrobial activity, LAB-derived compounds may affect several traits involved in bacterial persistence and adaptation, including adhesion, motility, biofilm formation, and virulence-associated phenotypes (Che et al. 2024). Importantly, such effects may occur at subinhibitory concentrations without a corresponding reduction in planktonic growth, suggesting biological activities that are not solely attributable to growth inhibition (Rotta et al. 2025a,b; Kostoglou et al. 2026).

*Lactobacillus acidophilus* ATCC 4356, originally isolated from human infant feces, is a well-characterized probiotic strain with properties relevant to microbial antagonism and host interactions (Palomino et al. 2015). Its probiotic potential has been associated with tolerance to gastrointestinal conditions, interactions with intestinal epithelial and immune cells, and antimicrobial activity against bacterial pathogens. More recent studies have further demonstrated biologically relevant properties of *L. acidophilus*, including modulation of intestinal epithelial barrier function (Haque et al. 2024), and antimicrobial effects mediated by secreted compounds (Aghmiyuni et al. 2024).

Despite the recognized antimicrobial potential of LAB-derived metabolites, the effects of the cell-free supernatant produced by *L. acidophilus* ATCC 4356 on biofilm formation, motility, and associated regulatory responses in *L. monocytogenes* remain poorly understood. Therefore, the present study investigated the antimicrobial, antibiofilm, and antimotility effects of the cell-free supernatant derived from *L. acidophilus* ATCC 4356 (*La*-CFS) against *L. monocytogenes*. We first determined the inhibitory activity of *La*-CFS against multiple *L. monocytogenes* strains and subsequently evaluated the effects of subinhibitory concentrations on biofilm formation and swimming and swarming motility. In addition, extracellular signaling activity was assessed using an AI-2-responsive reporter system, and the transcriptional responses of selected QS- and motility-associated genes were examined to further characterize the responses of *L. monocytogenes* to *La*-CFS exposure.

## Materials and methods

### Bacterial strains and culture conditions

*L. acidophilus* ATCC 4356 was kindly provided by the Oswaldo Cruz Foundation (Fiocruz, Brazil). The strain was cultivated in de Man, Rogosa and Sharpe (MRS) broth (Kasvi, Spain) at 37 °C for 18–24 h under microaerophilic conditions. *L. monocytogenes* ATCC 19112, along with 20 strains isolated from food or food processing environments, were provided by the *Listeria* Collection (CLIST/FIOCRUZ) of the Bacterial Zoonoses Laboratory of the Oswaldo Cruz Foundation (LABZOO/IOC/Fiocruz) (Table 1). The *L. monocytogenes* isolates had previously been submitted to biochemical identification, molecular confirmation, and PCR serotyping. Biochemical identification was performed based on the fermentation of the carbohydrates alpha-methyl-D-mannoside, D-xylose, D-rhamnose, D-mannitol, and D-glucose, as well as the catalase test and hemolysis on 5% sheep blood agar (Merck, USA) (Rocourt et al. 1983a, b). PCR assay was performed targeting the 23 S rDNA region and the *hly* gene (Hudson, 2001), serotyping was determined using the PCR method described by Doumith et al. (2004a, b).

**Table 1:** *Listeria monocytogenes* strains used in the study. Identification, serotypes, years of isolation, Brazilian states of origin**,** and sources are shown

Nº CLIST	Scientific name	Year of isolation	State	Type	Origin
706	*Listeria monocytogenes* 4 b	2011	SP	Food	Yakisoba
1018	*Listeria monocytogenes* 4 b	2011	SP	Food	Mini pizza
1071	*Listeria monocytogenes* 4 b	2009	RS	Food	Powdered milk
3492	*Listeria monocytogenes* 4 b	2013	RS	Environment	Meat tenderizer
3803	*Listeria monocytogenes* 4 b	2014	BA	Food	Organic vegetables (beetroot)
3833	*Listeria monocytogenes* 1/2 b	2014	RS	Environment	Industrial floor
3837	*Listeria monocytogenes* 4 b	2014	RS	Environment	Drain in the processing room
3839	*Listeria monocytogenes* 1/2 b	2014	RS	Environment	Drain in the processing room
3992	*Listeria monocytogenes* 1/2 b	2015	RJ	Food	Chicken liver pâté
4001	*Listeria monocytogenes* 1/2c	2015	DF	Food	Beef
4107	*Listeria monocytogenes* 1/2 a	2015	RJ	Food	Temaki Philadelphia
4251	*Listeria monocytogenes* 1/2 b	2016	BA	Food	Packed chicken drumstick
4313	*Listeria monocytogenes* 1/2 b	2016	SP	Environment	Swab from dough preparation drain
4330	*Listeria monocytogenes* 1/2 b	2016	SP	Environment	Swab from Argentine slicer belt
4449	*Listeria monocytogenes* 1/2 a	2017	MT	Environment	Swab from boards and knives
4455	*Listeria monocytogenes* 1/2 a	2017	MT	Food	Frozen chicken carcass
4484	*Listeria monocytogenes* 1/2c	2017	MT	Food	Frozen chicken cuts
4506	*Listeria monocytogenes* 4 b	2017	RS	Environment	Conveyor table
4511	*Listeria monocytogenes* 1/2 b	2017	RS	Environment	Roller
4631	*Listeria monocytogenes* 1/2 b	2018	SC	Food	Ready-to-eat processed meat products

The *L. monocytogenes* strains were grown in Brain Heart Infusion (BHI) broth (Himedia, India) at either 28 °C or 37 °C, depending on the experimental design, for 18–24 h under aerobic conditions. For long-term storage, cultures were maintained at − 80 °C in BHI supplemented with 20% glycerol.

### *L. acidophilus* ATCC 4356 cell-free supernatant preparation and antimicrobial activity evaluation

Stationary-phase cultures of *L. acidophilus* ATCC 4356 were centrifuged at 12.500 g for 15 min, and supernatants were filtered through a 0.22 μm syringe filter to obtain cell-free supernatant (*La*-CFS). This was aliquoted into sterile 1.5 mL microtubes and stored at 4 °C until use.

The minimum inhibitory concentration (MIC) of *La*-CFS against *L. monocytogenes* strains was determined using the broth microdilution method (CLSI 2023). Serial two-fold dilutions of *La*-CFS (ranging from 50% to 3.125% (v/v) final concentration into the well) prepared in BHI broth were mixed to a standardized *L. monocytogenes* suspension (10^6^ CFU/mL; 10 μL) (resulting in a final volume of 100 µL per well). The microplates were incubated aerobically at 37 °C for 24 h. Control wells included BHI only, *La*-CFS only, and untreated *L. monocytogenes* cultures. The MIC was defined as the lowest concentration of *La*-CFS at which no visible bacterial growth was observed after 24 h of incubation. All assays were performed in biological triplicate and technical duplicate. The MIC was subsequently used as the reference for preparing subinhibitory concentrations, with 0.5×, 0.25 ×, and 0.125 × MIC corresponding to final *La*-CFS concentrations of 25%, 12.5%, and 6.25% (v/v), respectively.

Additionally, growth kinetics of *L. monocytogenes* CLIST 3833 were evaluated by monitoring OD_600_ over 24 h at 37 °C under static conditions, in the presence of inhibitory and subinhibitory concentrations of *La*-CFS. The assay was performed in three independent biological experiments, each with three technical replicates per treatment. OD_600_ measurements were obtained from the same cultures at predetermined time points.

### Amplification and analysis of the *luxS *gene of *L. monocytogenes* strains

Genomic DNA from *L. monocytogenes* strains was extracted using the phenol–chloroform method (Gautam 2022). The DNA was precipitated with absolute ethanol, washed with 70% ethanol, air-dried, and resuspended in ultrapure water. DNA concentration was determined using a Thermo Scientific NanoDrop Lite Spectrophotometer. The *luxS* gene was amplified by conventional PCR using a commercial Master Mix kit (Applied Biosystems, Thermo Fisher Scientific). Each reaction contained 45 µL of Master Mix, each primer at a final concentration of 200 nM (Forward: GGAAAT GCC AGC GCT ACA CTC TTT; Reverse: ATT GCA TGC AGG AACTTC TGT CGC) (Thorat et al. 2019), 50 ng of genomic DNA, and nuclease-free water to a final volume of 50 µL. The amplification was performed on a GeneAmp™ PCR System 9700 (Applied Biosystems) using the following conditions: 94 °C for 2 min, 30 cycles of 94 °C for 30 s, 58 °C for 30 s, and 72 °C for 1 min. PCR products were analyzed on agarose gel (1.5% w/v) stained with ethidium bromide, alongside the Invitrogen™ 100 bp DNA Ladder and visualized under UV light to confirm amplification. The expected size of the *luxS* gene fragment is 208 bp (Thorat et al. 2019).

### AI-2 activity detection using *Vibrio harveyi* BB170

Among the 21 L*. monocytogenes* strains evaluated, 14 had previously been classified as strong biofilm producers (Rotta et al. 2025b). Of these, CLIST 3833, CLIST 3837, CLIST 3839, and CLIST 4001 were selected for the AI-2 functional assay and subsequent phenotypic analyses, because they exhibited more consistent experimental responses, characterized by lower variability among replicates and greater reproducibility of biofilm formation and *La*-CFS-induced phenotypic effects across experiments. Considering the established relationship between biofilm development and quorum sensing-associated regulatory mechanisms in *L. monocytogenes*, these strains were selected as representative isolates exhibiting a robust biofilm-forming phenotype for further investigation of AI-2 activity and the effects of *La*-CFS on QS-associated phenotypes.

AI-2 signaling activity was evaluated using the bioluminescent reporter strain *Vibrio harveyi* BB170, as described by Bassler et al. (1997), with adaptations. *V. harveyi* BB170 biosensor strain specifically detects the exogenous AI-2 molecule, inducing luminescence. *V. harveyi* BB170 was activated in Autoinducer Bioassay (AB) medium and incubated at 30 °C with continuous shaking (200 g). *V. harveyi* BB170 overnight culture was diluted 1:5000 in fresh AB medium. For detection of AI-2 production, cell-free culture fluid (CF; 100 μL) obtained by filtering the supernatant of *L. monocytogenes* cultures (grown in BHI broth, for 24 h, at 37 °C) through a 0.22 µm-pore size filter (Kasvi) was mixed to 900 μL of the *V. harveyi* BB170 suspension in sterile Eppendorf tubes. The mixture was vortexed and incubated at 30 °C with continuous stirring (200 g). The CF from *Escherichia coli* DH5α was used as negative control for AI-2 production (Taga and Xavier 2011).

Bioluminescence was measured over 7 h using the EasyTox ET-400 luminometer workstation (Labtest, Brazil). The EasyTox platform is designed for ecotoxicological bioassays and is equipped with a luminometer, incubation module (EasyCool), and EasyData® software for automated acquisition and processing of luminescence data, enabling sensitive detection of bioluminescent responses. In this study, the system was adapted for monitoring AI-2 activity through *V. harveyi* BB170 luminescence.

Bioluminescent activity was measured in relative light units (RLU) (Taga and Xavier 2011), and calculated for each treatment at each time point as follows:

RLU = (mean luminescence of treatment) / (mean luminescence of negative control at the same timepoint).

All assays were performed in biological triplicates with technical duplicates.

### Biofilm inhibition assay

The antibiofilm activity of *La*-CFS against *L. monocytogenes* strains was evaluated in sterile, flat-bottomed 96-well polystyrene microplates, using the crystal violet (CV) staining method (Liu et al. 2021, with modifications). Each well received 50 μL of double-concentrated BHI broth (2x), 50 μL of *La*-CFS at subinhibitory concentrations (0.5 × MIC, 0.25 × MIC, 0.125 × MIC), and 10 μL of *L. monocytogenes* suspension (10^6^ CFU/mL). After incubation, at 28 °C for 72 h, the microplate wells were washed twice with sterile saline (0.9%) to remove planktonic cells. Subsequently, crystal violet solution (0.25%, 100 μL) was added to each well and the microplates were incubated at room temperature for 30 min. Excess dye was removed by rinsing the wells with sterile distilled water, and the bound stain was solubilized with ethanol (95%, 100 μL). The absorbance was measured at 540 nm using a microplate reader (Kasvi, K-12096).

Wells containing *L. monocytogenes* non-exposed to *La*-CFS were used as positive controls (100% biofilm formation), and sterile medium without bacterial inoculum as the negative control. All assays were performed in biological triplicates with technical duplicates. Results were expressed as mean ± standard deviation.

### Motility inhibition assays

The effect of *La*-CFS on *L. monocytogenes* motility was assessed through swimming and swarming assays (Liu et al. 2021). The medium to evaluate swimming motility contained 1% tryptone, 0.5% NaCl, and 0.3% agar. Swarming motility was evaluated on plates containing 3% tryptone soy broth (TSB) and 0.5% agar (w/v). *La*-CFS was incorporated into the medium at subinhibitory concentrations (0.5 × MIC, 0.25 × MIC, and 0.125 × MIC). Overnight cultures of *L. monocytogenes* adjusted to 10^6^ CFU/mL were inoculated (5 µL) at the center of the agar plates containing the respective motility media. Plates were incubated at 28 °C for 48 h, and the colony diameters were measured in millimeters.

The motility percentage was calculated relative to the untreated control (100% motility), according to the following equation:

Motility (%) = (motility of the treated sample/motility of the untreated control) × 100.

All motility assays were conducted in triplicate.

### Influence of *La*-CFS on *L. monocytogenes* gene expression

The effect of *La*-CFS on the expression of genes related to quorum sensing (*luxS* and *agrC*) and motility (*flaA* and *motA*) was evaluated by real-time quantitative reverse transcription polymerase chain reaction (RT-qPCR) (Table 2). *L. monocytogenes* CLIST 3833 cultures (10^6^ CFU/mL), and 0.5 × MIC of *La*-CFS were mixed and incubated at 28 °C for 24 h. Following incubation, cultures were centrifuged (12.500 g, 5 min.), washed with PBS (1 M, pH 7.0), and total RNA was extracted using Trizol reagent (Sigma-Aldrich, USA). The RNA concentration and purity were determined spectrophotometrically using a NanoDrop 2000 (Thermo Fisher Scientific, USA). RNA integrity was verified by agarose gel electrophoresis, and contaminating genomic DNA was removed by DNase I treatment (Invitrogen, USA).

**Table 2:** Primers used in real-time quantitative polymerase chain reactions (RT-qPCR)

Gene	Primer name	Primer sequence (5’–3’)	References
*16 S rRNA*	16 S-F 16 S-R	ACCGTCAAGGGACAGCA GGGAGGCAGCAGTAGGGA	Liu et al. (2021)
*luxS*	luxS-F luxS-R	AAGCACCTTTTGTGAGACTGG CCGTTAGTGTTGTAGCGATGA	Pieta et al. (2017)
*agrC*	agrC-F agrC-R	GGGGTCAATCGCAGGTTTTG CTTTAAGTTCGTTGGTTGCCGTA	Liu et al. (2021)
*flaA*	flaA-F flaA-R	GGCTGCTGAAATGTCCGAAA TGCGGTGTTTGGTTTGCTTG	Pieta et al. (2017)
*motA*	motA-F motA-R	TTTTACGGGATGTTTTGGAA TCGCTAAGTTTGTCTGGGTT	Pieta et al. (2017)

Complementary DNA (cDNA) was synthesized with the High-Capacity cDNA Reverse Transcription Kit (Thermo Fisher, USA), following the manufacturer’s recommendations. RT-qPCR reactions were carried out in a StepOnePlus Real-Time PCR System (Applied Biosystems, USA) and performed with PowerTrack™ SYBR Green Master Mix for qPCR (Thermo Fisher, USA), following the manufacturer’s instructions.

Each reaction mixture (10 μL) contained 5 μL of PowerTrack SYBR Green Master Mix, 0.5 μL of forward primer (10 µM stock; 500 nM final concentration), 0.5 μL of reverse primer (10 µM stock; 500 nM final concentration), 1 μL of cDNA (20 ng), and nuclease-free water to a final volume of 10 μL. Amplification conditions were adopted from the literature: initial denaturation at 95 °C for 2 min, followed by 40 cycles of 95 °C for 15 s and 60 °C for 60 s. A melting curve analysis was performed to confirm amplification specificity. Relative gene expression levels were calculated using the comparative CT method (2^−ΔΔCt^) (Shi et al. 2024; Livak and Schmittgen 2001). Expression values were normalized to the housekeeping gene *16 S rRNA*. All experiments were performed in biological duplicates and experimental triplicates.

### Statistical analyses

The statistical analyses were conducted using GraphPad Prism 8.0 software (GraphPad Software Inc., La Jolla, CA, USA). Growth kinetics data were analyzed using two-way repeated-measures analysis of variance (ANOVA), considering treatment and time as factors, followed by Dunnett’s multiple-comparisons test to compare each *La*-CFS concentration with the untreated control. Comparisons between *La*-CFS–treated groups and untreated controls were analyzed by one-way ANOVA followed by Dunnett’s multiple comparison test to evaluate the influence of *La*-CFS on biofilm formation and motility. Differences were considered statistically significant when *p* < 0.05. For AI-2 activity assays, luminescence values were normalized as relative luminescence units (RLU) and compared by two-way ANOVA followed by Dunnett’s test at each time point. For relative gene expression analysis, fold changes were calculated using the 2^ − ΔΔCt method. Genes were classified as upregulated (2^ − ΔΔCt > 1) or downregulated (2^ − ΔΔCt < 1) in the presence of *La*-CFS. All experiments were performed in at least two independent biological replicates, with technical duplicates for each condition.

## Results

### Antagonistic activity of *La*-CFS against *L. monocytogenes* strains

**Fig. 1:**
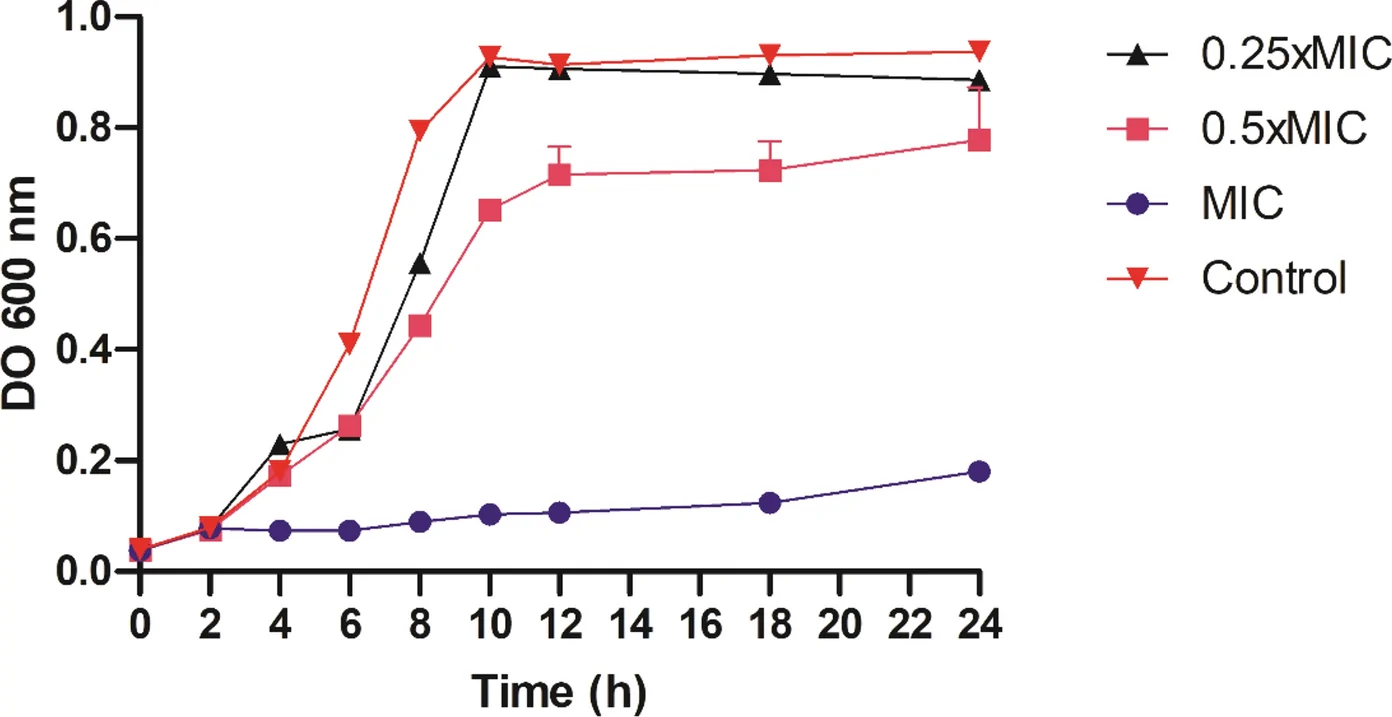
Growth kinetics of *Listeria monocytogenes* CLIST 3833 exposed to subinhibitory and inhibitory concentrations of *La*-CFS. Bacterial growth was monitored by measuring OD_600_ over 24 h in the absence of *La*-CFS (control) and in the presence of 0.25 × MIC, 0.5 × MIC, and MIC concentrations. Data are presented as mean ± SD of three independent replicates

The inhibitory activity of *La*-CFS was assessed by determining its MIC against all twenty-one *L. monocytogenes* strains. For all strains evaluated, the MIC corresponded to 50% *La*-CFS (v/v), defined as the lowest concentration at which no visible bacterial growth was observed. Growth-kinetic analysis of *L. monocytogenes* CLIST 3833 further demonstrated a concentration-dependent effect of *La*-CFS (Fig. 1). At the MIC, bacterial growth remained markedly suppressed throughout the first 18 h of incubation, followed by an increase in OD_600_ at later time points (*p* < 0.05). At subinhibitory concentrations, *La*-CFS induced a transient delay in bacterial growth, with significant differences in OD_600_ observed at 6 and 8 h for 0.25 × MIC and at 6, 8, and 10 h for 0.5 × MIC (*p* < 0.05), while no significant differences were detected at 24 h (*p* > 0.05). As substantial bacterial growth was maintained at both sub-MIC concentrations, 0.25 × and 0.5 × MIC were selected for subsequent phenotypic assays to evaluate the effects of *La*-CFS on biofilm formation and motility under conditions in which bacterial growth was largely preserved.

### Detecting AI-2 activity using *Vibrio harveyi* BB170 as a biosensor

**Fig. 2:**
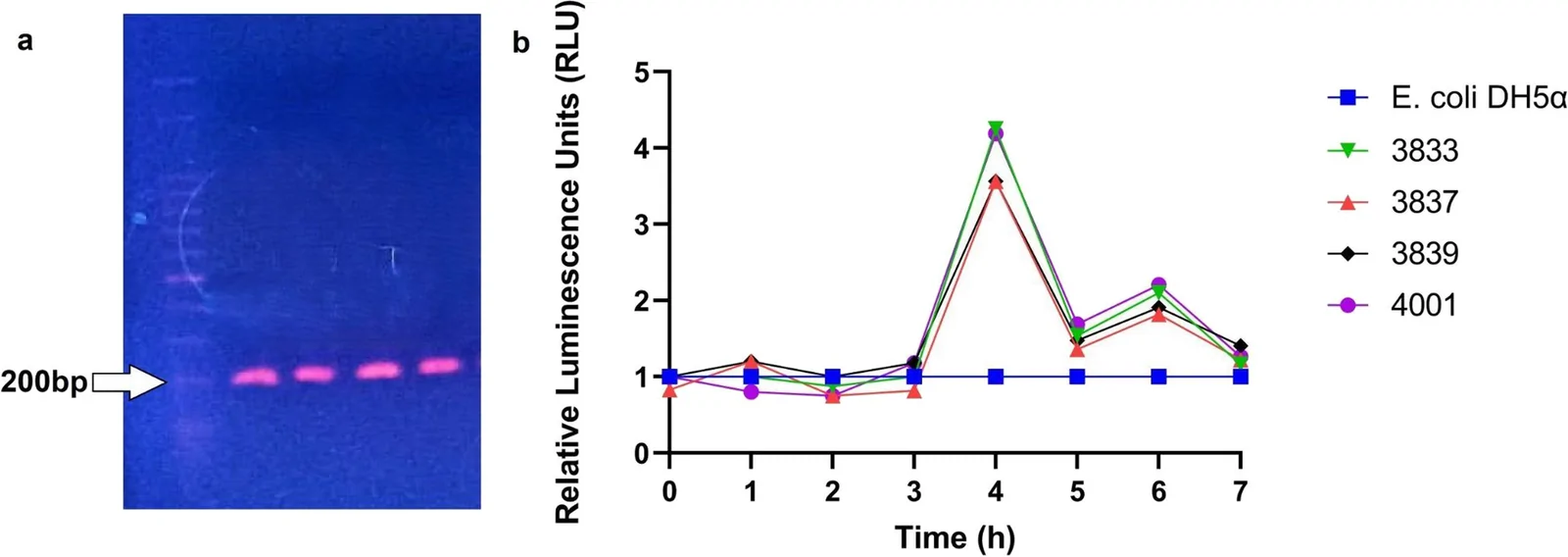
**a** Conventional PCR amplification of the *luxS* gene in *L. monocytogenes* strains. Lanes 1–4 correspond to strains CLIST 3833, 3837, 3839, and 4001, respectively. The presence of a band around 200 bp confirmed the presence of the AI-2 synthase gene in all evaluated strains (expected size of 208 bp). **b** Induction of bioluminescence (RLU) in *V. harveyi* BB170 over 7 h-exposure to the culture supernatants of *L. monocytogenes* strains (CLIST 3833, 3837, 3839, and 4001), indicating AI-2 autoinducer activity

The *luxS* gene was detected by conventional PCR in the four *L. monocytogenes* strains evaluated (Fig. 2a), indicating possible AI-2 production. To determine whether this genetic potential translated into functional signaling, AI-2 activity was evaluated using the bioluminescent reporter strain *Vibrio harveyi* BB170.

In the presence of the culture supernatants of *L. monocytogenes* strains CLIST 3833, CLIST 3837, CLIST 3839, and CLIST 4001, *V. harveyi* BB170 exhibited an increase in relative luminescence units (RLU) during the 7-h monitoring period. A **s**ubstantial rise in luminescence was observed at the 4-h time point, indicating the peak of AI-2 activity for all strains (RLU values ranging from 3.56 to 4.25). For comparison, the negative control *E. coli* DH5α, displayed an RLU value of 1.0 under the same conditions. The considerably higher RLU levels elicited by the *L. monocytogenes* supernatants demonstrate strong AI-2 signaling activity across all strains tested, with strains CLIST 3833 and CLIST 4001 showing the highest induction (Fig. 2b).

Based on their previous characterization as strong biofilm producers (Rotta et al. 2025b), strains CLIST 3833, CLIST 3837, CLIST 3839, and CLIST 4001 were selected for functional assessment of AI-2 activity.

### Biofilm formation

As recently reported by our research group, the strains of *L. monocytogenes* used in the present study are able to form biofilms on polystyrene microplates, and the strains CLIST 3833, 3837, 3839, and 4001 were classified as strong biofilm producers (Rotta et al. 2025b). Taking this phenotype into account, along with the enhanced induction of bioluminescence in *V. harveyi* BB170 (Fig. 2B), the effect of *La*-CFS on biofilm formation by the strains 3833, 3837, 3839, and 4001 was determined after 72 h of incubation at 28 °C.

**Fig. 3:**
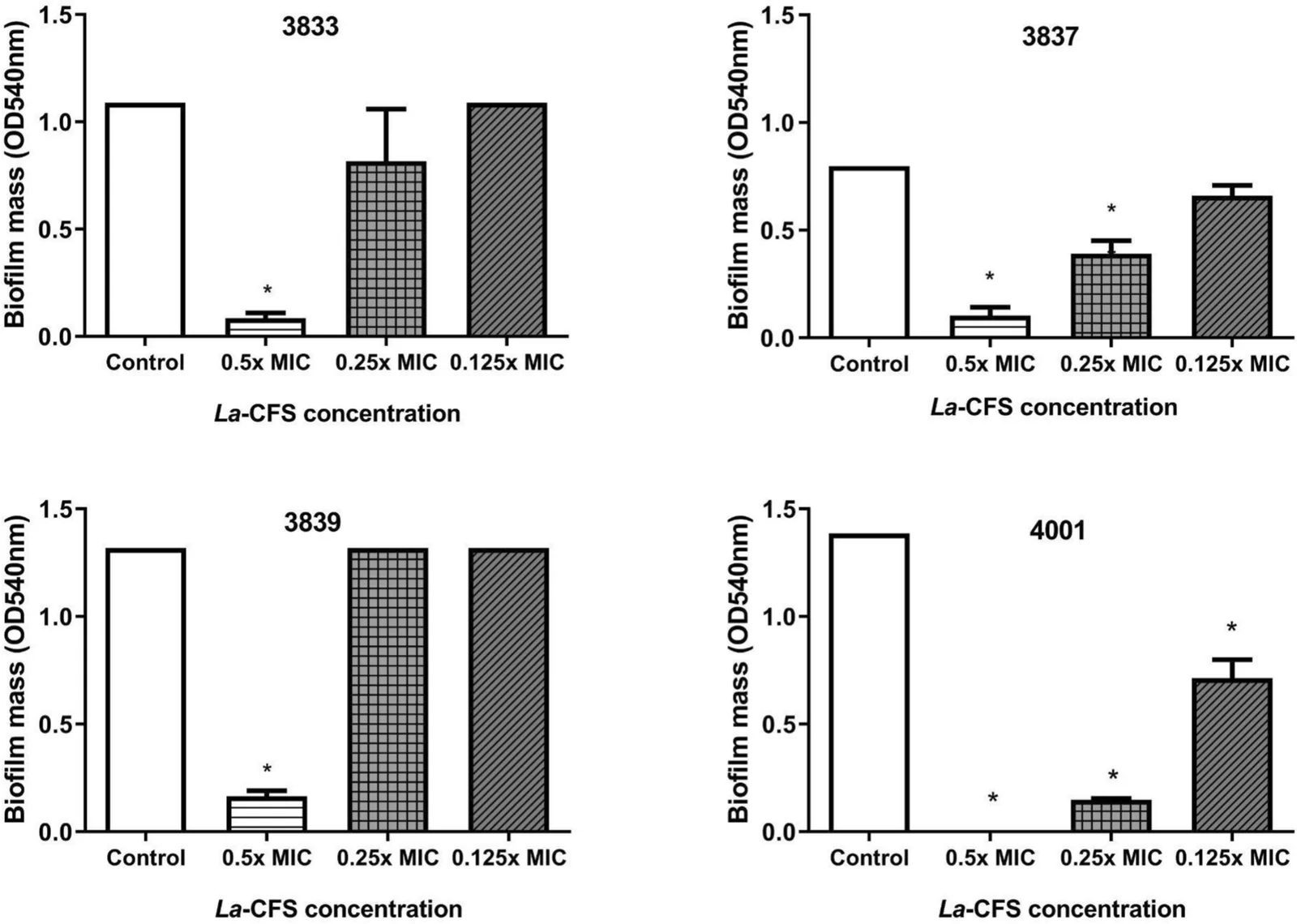
Influence of subinhibitory concentrations of *La*-CFS (0.5×, 0.25×, and 0.125 × MIC) on biofilm formation by *Listeria monocytogenes* strains (CLIST 3833, 3837, 3839, 4001 a-d). Crystal violet assay was used to determine biofilm biomass (absorbance at 540 nm). Control: biofilm formed in the absence of *La*-CFS. The asterisks indicate statistically significant differences when compared to the control (*p* < 0.05)

At sub-MIC, *La*-CFS significantly reduced biofilm biomass in a dose- and strain-dependent manner (Fig. 3). At 0.5 × MIC the treatment reduced the biofilm formation capacity of all *L. monocytogenes* strains evaluated (p < 0.05) (reduction rates varying from 86 to 100%). At concentrations below 0.5 × MIC, the antibiofilm activity of *La*-CFS was markedly strain-dependent, with significant inhibition observed only in selected strains. At 0.25 × MIC, the inhibitory effect also varied among strains, being more pronounced in strain CLIST 4001 (approximately 89% inhibition, *p* < 0.05) and strain CLIST 3837 (reduction of approximately 50% in biofilm formation). At 0.125 × MIC, *La*-CFS still prevents biofilm formation in strain CLIST 4001 (*p* < 0.05), demonstrating that even low concentrations can interfere with biofilm development (*p* < 0.05). Biofilm formation by the strains CLIST 3833 and 3839 was not affected by concentrations equal to or below 0.25 × MIC (*p* > 0.05) (Fig. 3).

### Swarming and swimming motility

Two main types of motilities can be detected in *L. monocytogenes* cells: swimming and swarming. Swimming motility represents the movement of single cells through liquid environments via flagellar rotation, whereas swarming motility involves the coordinated migration of cells across surfaces, driven by helical flagella and regulated by quorum sensing mechanism.

**Fig. 4:**
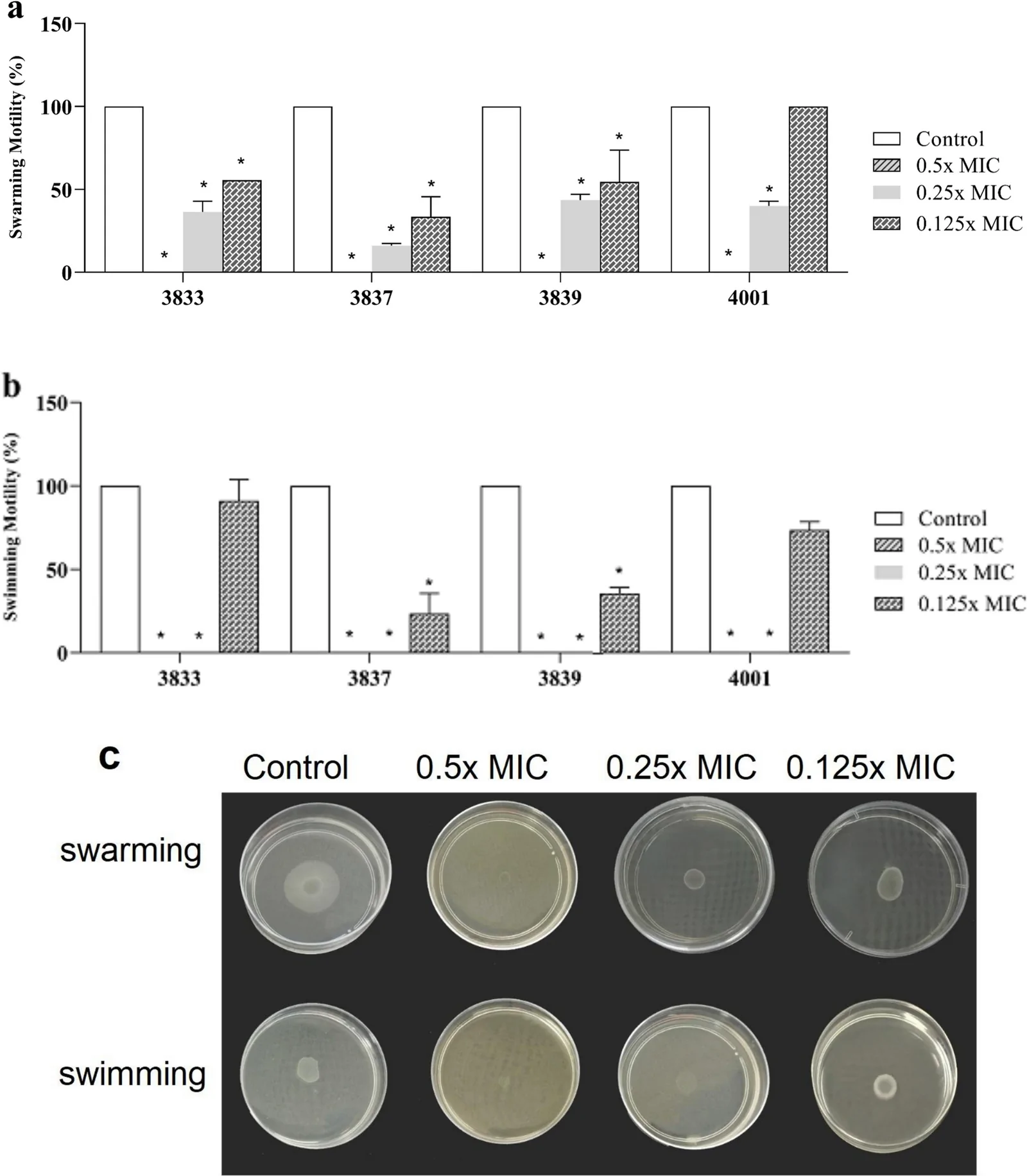
Efficacy of different concentrations of *Lactobacillus acidophilus* ATCC 4356 cell-free supernatant (0.5x, 0.25 × and 0.125 × MIC) in inhibiting motility of *Listeria monocytogenes* strains at 28 °C. (a) Swarming ability, (b) Swimming ability, (c) representative images of the *La*-CFS activity on motility patterns of *L. monocytogenes* strain CLIST 3833 at absence (control) and presence of 0.5–0.125 × MIC. Bars with an asterisk indicate significant differences compared to the control group (non-treated with *La*-CFS), according to Dunnett’s test (*p* < 0.05)

The effect of *La*-CFS on *L. monocytogene*s motility was evaluated in semi-solid agar assays, and the effect was also dose- and strain-dependent. The strains CLIST 3833, 3837, 3839, and 4001 consistently exhibited reduced motility under *La*-CFS treatment (Fig. 4). At 0.5 × MIC of *La*-CFS, swimming and swarming motility were completely inhibited. In the presence of 0.25 × MIC of *La*-CFS, the swarming motility of *L. monocytogenes* strains CLIST 3833, 3837, 3839, and 4001 showed a significant reduction compared to unexposed cells (reduction rates ranging from 59 to 85%) (Fig. 4a and c). The swimming motility of all four *L. monocytogenes* strains was affected by the presence of *La*-CFS (100% reduction in all strains at 0.5 × and 0.25 × MIC, and 33 to 85% at 0.125 × MIC) (Fig. 4b and c).

### Changes in gene expression induced by *La*-CFS

**Fig. 5:**
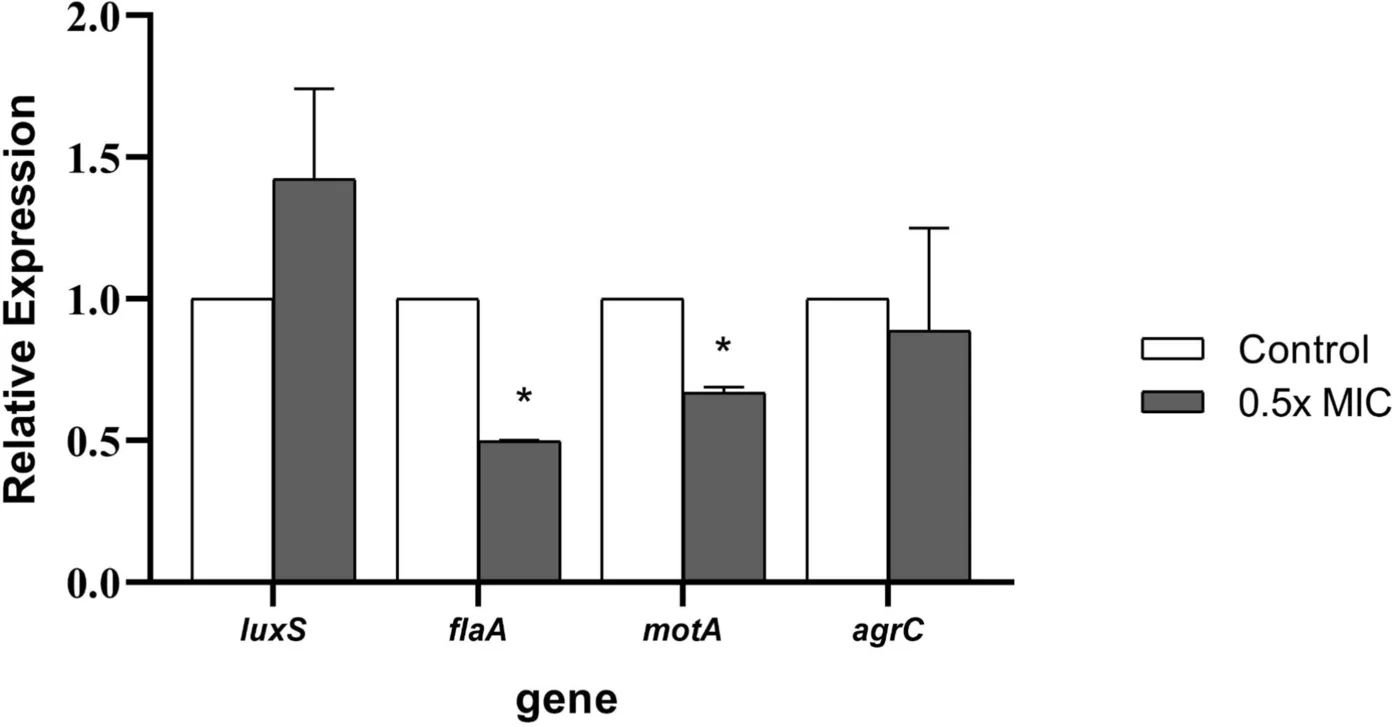
Relative expression of target genes in *L. monocytogenes* CLIST 3833 after 24-h exposure to 0.5 × MIC of *La*-CFS, at 28 °C for 24 h. (*) indicates statistically significant differences (*p* < 0.05) compared to the control group (not exposed to *La-*CFS). Bars represent the mean ± standard deviation of three independent experiments

Based on its consistent phenotypic response to *La*-CFS in the biofilm and motility assays, *L. monocytogenes* CLIST 3833 was selected for transcriptional analysis. Exposure to 0.5 × MIC of *La*-CFS for 24 h significantly downregulated the expression of the motility-related genes *flaA* and *motA* (*p* < 0.05), whereas no significant changes were observed in the expression of the QS-associated genes *agrC* and *luxS* (*p* > 0.05) (Fig. 5). These findings suggest that the inhibitory effect of *La*-CFS on motility may be associated, at least in part, with modulation of flagellar gene expression.

## Discussion

*L. acidophilus* ATCC 4356 is recognized for the production of a wide range of bioactive compounds, including lactic acid, hydrogen peroxide, exopolysaccharides, short-chain fatty acids, and bacteriocins. In this context, the present study demonstrates that the cell-free supernatant obtained from *L. acidophilus* ATCC 4356 (*La*-CFS) exerts antagonistic activity against *L. monocytogenes* and, at subinhibitory concentrations, modulates biofilm formation and motility, two phenotypes relevant to surface colonization and environmental persistence.

*La*-CFS was able to inhibit the growth of twenty-one *L. monocytogenes* strains isolated from food and food-processing environments and belonging to four different serotypes. The antimicrobial activity observed in the present study is consistent with previous reports demonstrating the inhibitory potential of metabolites produced by *L. acidophilus* ATCC 4356 against different bacterial species (Sarikhani et al. 2018; Aghmiyuni et al. 2024). Beyond this direct antimicrobial effect, the use of subinhibitory concentrations allowed us to investigate whether *La*-CFS could also modulate *L. monocytogenes* phenotypes without compromising bacterial growth. Indeed, sub-MIC levels of *La*-CFS did not significantly affect *L. monocytogenes* growth, supporting the interpretation that the changes observed in biofilm formation and motility were not simply secondary to growth inhibition.

Biofilm formation in *L. monocytogenes* is a multifactorial process shaped by environmental conditions, strain-specific characteristics, adhesion, motility, and regulatory networks, including QS-related systems (Riedel et al. 2009; Mazaheri et al. 2021). These coordinated traits contribute to surface colonization and persistence in food-processing environments. Once established, biofilms form structured multicellular communities in which bacterial cells are embedded within an extracellular polymeric matrix, providing protection against environmental stresses and increasing tolerance to antimicrobial agents (Prinzi and Rohde 2023; Liu et al. 2024).

Flagellum-mediated motility plays an important role in surface exploration, initial attachment, and biofilm development in *L. monocytogenes*. By enabling bacterial cells to reach and interact with surfaces, flagellar motility can help overcome hydrodynamic and repulsive forces that would otherwise hinder adhesion (Kerekes et al. 2013; Hossain et al. 2021; Vilas Boas et al. 2024). Experimental studies have shown that flagella and flagellum-mediated motility influence the initial attachment of *L. monocytogenes* to abiotic surfaces, including stainless steel, and contribute to subsequent biofilm development (Vatanyoopaisarn et al. 2000; Lemon et al. 2007; Todhanakasem and Young 2008). This motility may occur through distinct mechanisms, including swimming, which involves the movement of individual cells in liquid or semi-solid environments driven by flagellar rotation, and swarming, a coordinated multicellular movement across surfaces that can contribute to bacterial dissemination and early surface colonization (Kearns 2010; Lemon et al. 2007; Wadhwa and Berg 2021).

In the present study, exposure to sub-MIC levels of *La*-CFS significantly reduced biofilm formation by *L. monocytogenes* in a dose- and strain-dependent manner, with inhibition reaching up to 100% at 0.5 × MIC. The magnitude of this effect varied among strains, and some remained susceptible to *La*-CFS even at lower concentrations. These findings are consistent with previous studies from our research group demonstrating the antibiofilm potential of CFS produced by *Lacticaseibacillus rhamnosus* ATCC 9595 and *Weissella paramesenteroides* UFTM 2.6.1 against *L. monocytogenes* (Rotta et al. 2025a,b), and extend this evidence to the probiotic strain *L. acidophilus* ATCC 4356. Similar antibiofilm effects have also been reported for the CFS of *Faecalibacterium prausnitzii*, which markedly reduced *L. monocytogenes* biofilm biomass and affected other biofilm-associated traits (Wang et al. 2025).

Concentration-dependent inhibition of *L. monocytogenes* biofilm formation at sub-MIC levels has been described for natural antimicrobial compounds such as cinnamaldehyde, eugenol, resveratrol, and thymoquinone (Liu et al. 2021). Collectively, these findings support the notion that subinhibitory exposure to microbial metabolites and other bioactive compounds can modulate biofilm-associated phenotypes in *L. monocytogenes* without necessarily impairing bacterial growth. Because *La*-CFS was present throughout biofilm development, its inhibitory effect may involve interference with early stages of biofilm establishment, including motility and initial attachment.

Consistent with this possibility, *La*-CFS markedly impaired both swimming and swarming motility in *L. monocytogenes*. Complete inhibition was observed at 0.5 × and 0.25 × MIC, with substantial reductions even at lower concentrations, suggesting that motility-related functions are particularly sensitive to subinhibitory exposure to *La*-CFS. Similar effects have been reported for other bioactive agents. CFS produced by lactic acid bacteria has been shown to impair *L. monocytogenes* motility (Hossain et al. 2021a), whereas subinhibitory concentrations of glabridin significantly reduced both swimming and swarming in a concentration-dependent manner (Liao et al. 2024). Likewise, thymoquinone reduced *L. monocytogenes* motility at subinhibitory concentrations, an effect accompanied by decreased transcription of *flaA*, which encodes flagellin (Miao et al. 2019). Together, these observations support the possibility that the reduction in motility induced by *La*-CFS may contribute to its antibiofilm activity, although the molecular mechanisms underlying this association remain to be established.

To investigate the molecular changes associated with these phenotypic responses, the expression of *luxS, agrC, flaA,* and *motA* was analysed by RT-qPCR. Among the genes evaluated, *flaA* was significantly downregulated following *La*-CFS exposure, consistent with the pronounced impairment of swimming and swarming observed in the phenotypic assays. Given the important role of flagellum-mediated motility in surface exploration, initial attachment, and early biofilm development in *L. monocytogenes* (Lemon et al. 2007), reduced *flaA* expression may contribute to the concomitant decreases in motility and biofilm formation observed in this study, although a direct causal relationship cannot be established. Similar reductions in motility associated with decreased *flaA* expression have been reported following exposure of *L. monocytogenes* to natural antimicrobial compounds, including cinnamaldehyde, eugenol, and thymoquinone (Upadhyay et al. 2013; Miao et al. 2019).

Flagellar motility, however, depends not only on structural components such as flagellin but also on the activity of the flagellar motor, which includes the MotA and MotB proteins (Chen et al. 2011). In the present study, *motA* was also significantly downregulated following *La*-CFS exposure, indicating that the transcriptional response extended to multiple components of the motility machinery. Comparable transcriptional and phenotypic responses have been reported following exposure of *L. monocytogenes* to other bioactive compounds. Sublethal exposure to *Cannabis sativa* essential oil reduced the expression of *flaA, motA,* and *motB* and was accompanied by impaired motility (Marini et al. 2018), while photoactivated luteolin reduced the expression of *motA* and *motB*, together with decreases in motility and biofilm formation (Feng et al. 2026). Pyrrole − 2-carboxylic acid similarly impaired swimming and swarming motility and adhesion while downregulating genes involved in flagellar assembly and chemotaxis (Jiang et al. 2025a). Importantly, the expression and regulation of motility-associated genes may vary according to environmental conditions and bacterial genetic background (Mattila et al. 2011). Inter-strain differences have also been reported for *motA* and *motB*, with basal expression varying among sequence types and genetic lineages of *L. monocytogenes* (Gao et al. 2024). Such variability may contribute to differences in the magnitude of the phenotypic response to *La*-CFS among strains.

In addition to motility-associated genes, QS-related targets were investigated because regulatory systems involved in intercellular signaling have been implicated in *L. monocytogenes* biofilm development. The *luxS* gene, which is involved in AI-2 synthesis, is highly conserved in *L. monocytogenes* and has been detected across different strains and serotypes, suggesting that AI-2 production is widespread within the species (Surette et al. 1999; Sela et al. 2006; Challan Belval et al. 2006; Garmyn et al. 2009; Gao et al. 2024). However, the relationship between LuxS, AI-2, and biofilm formation appears to be complex, since *luxS*-deficient mutants have shown increased attachment and biofilm formation, whereas exogenous AI-2 did not restore the parental biofilm phenotype (Sela et al. 2006; Challan Belval et al. 2006). In the present study, all four *L. monocytogenes* strains evaluated (CLIST 3833, 3837, 3839, and 4001) harbored the *luxS* gene, and their cell-free supernatants induced bioluminescence in the *V. harveyi* BB170 reporter assay. These findings indicate the presence of extracellular signaling activity detectable by an AI-2-responsive reporter and are consistent with the production of AI-2-like signaling molecules by the evaluated strains.

Despite the consistent effects of *La*-CFS on motility- and biofilm-associated phenotypes, these responses cannot be specifically attributed to disruption of quorum sensing (QS), since no significant changes were detected in *luxS* or *agrC* expression under the conditions evaluated. In contrast, the significant downregulation of *flaA* and *motA*, together with the marked impairment of swimming and swarming motility, indicates a more pronounced effect of *La*-CFS on motility-associated responses. Nevertheless, the absence of significant transcriptional changes in *luxS* and *agrC* does not exclude the possibility of QS interference through other regulatory or signaling mechanisms. Moreover, because gene expression was assessed only after 24 h of exposure, transient transcriptional changes occurring at earlier stages cannot be ruled out. Time-resolved analyses, together with approaches targeting QS signaling beyond gene transcription, will therefore be important to further elucidate the regulatory mechanisms associated with the phenotypic effects of *La*-CFS.

In contrast to the transcriptional pattern observed for *La*-CFS, several antimicrobial compounds have been shown to affect QS-related pathways in *L. monocytogenes*. Subinhibitory concentrations of cinnamaldehyde, eugenol, resveratrol, and thymoquinone were reported to reduce biofilm formation while significantly downregulating *agrA, agrC,* and *agrD* (Liu et al. 2021). Cinnamaldehyde has also been shown to suppress transcription of the *agr* operon and interfere with AgrA-mediated regulation (Jiang et al. 2023). Similarly, licochalcone A reduced *luxS* expression and AI-2 signaling activity concomitantly with decreases in biofilm formation and motility (Jiang et al. 2025b). Thus, although these agents and *La*-CFS produce comparable effects on motility and biofilm formation, their transcriptional responses differ, highlighting the possibility that similar phenotypic outcomes may arise through distinct regulatory pathways and underscoring the complexity of the mechanisms governing *L. monocytogenes* biofilm development and persistence.

An important limitation of this study is that the bioactive constituents of *La*-CFS were not chemically characterized. Consequently, the observed effects cannot currently be attributed to a specific metabolite or class of compounds, and potential additive or synergistic interactions among different metabolites cannot be excluded. Furthermore, the potential influence of acidity on both the observed biological responses and the activity of *La*-CFS constituents should be considered. Further studies aimed at identifying and characterizing the bioactive molecules present in *La*-CFS, together with appropriate pH controls and time-resolved mechanistic analyses, are therefore needed to clarify the molecular basis of the antimicrobial, antibiofilm, and motility-related effects observed in *L. monocytogenes*.

### Concluding comments

The novelty of this study lies in demonstrating that metabolites produced by the well-characterized probiotic *L. acidophilus* ATCC 4356 can modulate biofilm formation and motility in the foodborne pathogen *L. monocytogenes*. At subinhibitory concentrations, *L. acidophilus* ATCC 4356 cell-free supernatant (*La*-CFS) markedly impaired both phenotypes while inducing only a transient delay in bacterial growth, without significantly affecting the final optical density, suggesting potential interference with processes involved in surface colonization and environmental persistence. These phenotypic effects were accompanied by significant downregulation of the motility-associated genes *flaA* and *motA*. However, they cannot be specifically attributed to disruption of quorum sensing, since no significant changes in *luxS* or *agrC* expression were detected under the conditions evaluated. Overall, these findings highlight the potential of *L. acidophilus*-derived metabolites as a source of bioactive compounds for controlling *L. monocytogenes* and reducing its persistence in food-processing environments. Further studies are needed to identify the bioactive constituents of *La*-CFS and to clarify the molecular mechanisms underlying its antimicrobial, antibiofilm, and motility-related effects.

## Data Availability

Data availability statement (restricted access) The datasets generated and analyzed during this study are available from the corresponding author but are not publicly available due to institutional and research restrictions. The data were generated specifically for this study and are available upon reasonable request, with permission from the responsible research institution.

## References

[CR1] Abedin MM, Chourasia R, Phukon LC, Sarkar P, Ray RC, Singh SP, Rai AK (2024) Lactic acid bacteria in the functional food industry: biotechnological properties and potential applications. Crit Rev Food Sci Nutr 64:10730–10748. 10.1080/10408398.2023.2227896 37405373

[CR2] Aghmiyuni ZF, Saderi H, Owlia P, Saidi N (2024) Evaluation of the effect of *Lactobacillus acidophilus* ATCC 4356 bacteriocin against *Staphylococcus aureus*. BioMed Res Int 2024:4119960. 10.1155/2024/4119960 PMC10980545 38559901

[CR3] Banicod RJS, Tabassum N, Javaid A, Kim YM, Khan F (2026) Lactic acid bacteria-derived secondary metabolites: emerging natural alternatives for food preservation. Probiotics Antimicrob Proteins 18:3113–3150. 10.1007/s12602-025-10672-6 40751876

[CR4] Bassler BL (1999) How bacteria talk to each other: regulation of gene expression by quorum sensing. Curr Opin Microbiol 2:582–587. 10.1016/S1369-5274(99)00025-9 10607620

[CR5] Bassler BL, Greenberg EP, Stevens AM (1997) Cross-species induction of luminescence in the quorum-sensing bacterium *Vibrio harveyi*. J Bacteriol 179:4043–4045. 10.1128/jb.179.12.4043-4045.1997 PMC179216 9190823

[CR6] Challan Belval S, Gal L, Margiewes S, Garmyn D, Piveteau P, Guzzo J (2006) Assessment of the roles of LuxS, S-ribosyl homocysteine, and autoinducer 2 in cell attachment during biofilm formation by *Listeria monocytogenes* EGD-e. Appl Environ Microbiol 72:2644–2650. 10.1128/AEM.72.4.2644-2650.2006 PMC1449078 16597969

[CR7] Che J, Shi J, Fang C, Zeng X, Wu Z, Du Q, Tu M, Pan D (2024) Elimination of pathogen biofilms via postbiotics from lactic acid bacteria: a promising method in food and biomedicine. Microorganisms 12:704. 10.3390/microorganisms12040704 PMC11051744 38674648

[CR8] Chen S, Beeby M, Murphy GE et al (2011) Structural diversity of bacterial flagellar motors. EMBO J 30:2972–2981. 10.1038/emboj.2011.186 PMC3160247 21673657

[CR9] CLSI (2023) Performance standards for antimicrobial susceptibility testing, 33rd edn. Clinical and Laboratory Standards Institute, Wayne, PA

[CR10] Doumith M, Buchrieser C, Glaser P, Jacquet C, Martin P (2004a) Differentiation of the major *Listeria monocytogenes* serovars by multiplex PCR. J Clin Microbiol 42(8):3819–3822. 10.1128/JCM.42.8.3819-3822.2004 PMC497638 15297538

[CR11] Doumith M, Buchrieser C, Glaser P, Jacquet C, Martin P (2004b) Differentiation of the major *Listeria monocytogenes* serovars by multiplex PCR. J Clin Microbiol 42:3819–3822. 10.1128/JCM.42.8.3819-3822.2004 PMC497638 15297538

[CR12] Federle MJ, Bassler BL (2003) Interspecies communication in bacteria. J Clin Invest 112:1291–1299. 10.1172/JCI20195 PMC228483 14597753

[CR13] Feng Y, Yang Y, Zhu Y, Zhao S, Mohd KK, Li C, Jin J, Zhang L, Tong S, Wu Y (2026) Investigation on enhanced antimicrobial activity and bactericidal mechanism of photoactivated luteolin against *Listeria monocytogenes*. Innov Food Sci Emerg Technol 108:104414. 10.1016/j.ifset.2025.104414

[CR14] Finn L, Onyeaka H, O’Neill S (2023) *Listeria monocytogenes* biofilms in food-associated environments and their control with natural products: a review. Foods 12:3339. 10.3390/foods12183339 PMC10529182 37761048

[CR15] Gao B, Cai H, Xu B, Yang F, Dou X, Dong Q, Yan H, Bu X, Li Z (2024) Growth, biofilm formation, and motility of *Listeria monocytogenes* strains isolated from food and clinical samples located in Shanghai (China). Food Res Int 184:114232. 10.1016/j.foodres.2024.114232 38609218

[CR16] Garmyn D, Gal L, Lemaitre J-P, Hartmann A, Piveteau P (2009) Communication and autoinduction in the species *Listeria monocytogenes*: a central role for the agr system. Commun Integr Biol 2:371–374. 10.4161/cib.2.4.8610 PMC2734052 19721895

[CR17] Gautam A (2022) Phenol-chloroform DNA isolation method. In: DNA and RNA isolation techniques for non-experts. Techniques in life science and biomedicine for the non-expert. Springer, Cham. 10.1007/978-3-030-94230-4_3

[CR18] Guerreiro DN, Wu J, Dessaux C, Oliveira AH, Tiensuu T, Gudynaite-Savitch L, Bai X, Lerminiaux NA, Libby SJ, Wallrodt I, Gray TA, de Carvalho CC, Fagerlund A, Bastos MC, Francke C, Riedel CU, Knudsen GM, Lunguya O, Johannessen M, Roop RM 2nd, Tang CM, Gerlach RG, Johansson J, Winkler J, McLennan AG, Ríos-Velázquez C, Camilli A, Mandin P, O’Byrne CP (2022) In vitro evolution of *Listeria monocytogenes* reveals selective pressure for loss of SigB and AgrA function at different incubation temperatures. Appl Environ Microbiol 88:e00330-e422. 10.1128/aem.00330-22 PMC9195950 35583325

[CR19] Haque M, Kaminsky L, Abdulqadir R, Engers J, Kovtunov E, Rawat M, Al-Sadi R, Ma TY (2024) *Lactobacillus acidophilus* inhibits the TNF-α-induced increase in intestinal epithelial tight junction permeability via a TLR-2 and PI3K-dependent inhibition of NF-κB activation. Front Immunol 15:1348010. 10.3389/fimmu.2024.1348010 PMC11286488 39081324

[CR20] Hossain MI, Kim K, Mizan MFR, Toushik SH, Ashrafudoulla M, Roy PK, Ha SD (2021) Comprehensive molecular, probiotic, and quorum-sensing characterization of anti-listerial lactic acid bacteria, and application as bioprotective in a food (milk) model. J Dairy Sci 104(6):6516–6534. 10.3168/jds.2020-19034 33741164

[CR21] Hossain MI, Mizan MFR, Roy PK, Nahar S, Toushik SH, Ashrafudoulla M et al (2021a) *Listeria monocytogenes* biofilm inhibition on food contact surfaces by application of postbiotics from *Lactobacillus curvatus* B.67 and *Lactobacillus plantarum* M.2. Food Res Int 148:110595. 10.1016/j.foodres.2021.110595. (**ª**) 34507740

[CR22] Hudson JA, Lake RJ, Savill MG, Scholes P, McCormick RE (2001a) Rapid detection of *Listeria monocytogenes* in ham samples using immunomagnetic separation followed by polymerase chain reaction. J Appl Microbiol 90(4):614–621. 10.1046/j.1365-2672.2001.01287.x 11309074

[CR23] Hudson JA, Lake RJ, Savill MG, Scholes P, McCormick RE (2001b) Rapid detection of *Listeria monocytogenes* in ham samples using immunomagnetic separation followed by polymerase chain reaction. J Appl Microbiol 90:614–621. 10.1046/j.1365-2672.2001.01287.x 11309074

[CR24] Jiang X, Kang R, Yu T, Jiang X, Chen H, Zhang Y, Li Y, Wang H (2023) Cinnamaldehyde targets the LytTR DNA-binding domain of the response regulator AgrA to attenuate biofilm formation of *Listeria monocytogenes*. Microbiol Spectr 11:e00300-23. 10.1128/spectrum.00300-23 PMC10269664 37140461

[CR25] Jiang L, Yue Y, Zhong K, Wu Y, Gao H (2025a) Antibiofilm mechanism of pyrrole-2-carboxylic acid by impairing flagellar motility of *Listeria monocytogenes* and its application in cheese. Food Control 168:110974. 10.1016/j.foodcont.2024.110974

[CR26] Jiang L, Han Y, Li X, Zhong K, Wu Y, Gao H (2025b) Inhibitory effect of licochalcone A on dual-species biofilms of *Listeria monocytogenes* and *Staphylococcus aureus*: mechanism involved in quorum sensing and application in beef. Int J Food Microbiol 442:111376. 10.1016/j.ijfoodmicro.2025.111376 40763449

[CR27] Kearns DB (2010) A field guide to bacterial swarming motility. Nat Rev Microbiol 8:634–644. 10.1038/nrmicro2405 PMC3135019 20694026

[CR28] Kerekes EB, Deák É, Tako M, Tserennadmid R, Petkovits T, Vágvölgyi C, Krisch J (2013) Anti-biofilm forming and quorum sensing inhibitory activity of selected essential oils and their main components on food-related microorganisms. J Appl Microbiol 115:933–942. 10.1111/jam.12289 23789847

[CR29] Kostoglou D, Vlachopoulou A, Vafeiadis G, Giaouris E (2026) Sustainable biofilm control using lactic acid bacteria to disrupt quorum sensing in foodborne pathogens. Sustain Food Technol 4:686–697. 10.1039/D5FB00384A

[CR30] Lemon KP, Higgins DE, Kolter R (2007) Flagellar motility is critical for *Listeria monocytogenes* biofilm formation. J Bacteriol 189:4418–4424. 10.1128/JB.01967-06 PMC1913361 17416647

[CR31] Li X, Wei G, Wang W, Liu H, Xin Q, Wang Z, Sun P, Ding T, Li J, Deng Y (2025) Mechanism insights into the regulation of the LuxS/AI-2 quorum sensing system on the formation of viable but nonculturable state in biofilm cells of beer-spoilage *Lactiplantibacillus plantarum*. J Adv Res 84:15–28. 10.1016/j.jare.2025.09.010 PMC13227230 40945846

[CR32] Liao C, Yu C, Guo J, Guan M (2024) Subinhibitory concentrations of glabridin from *Glycyrrhiza glabra* L. reduce motility and hemolytic activity of *Listeria monocytogenes* without exhibiting antimicrobial activity. Front Microbiol 15:1388388. 10.3389/fmicb.2024.1388388 PMC11288822 39086651

[CR33] Liu L, Wu R, Zhang J, Li P (2018) Overexpression of *luxS* promotes stress resistance and biofilm formation of *Lactobacillus paraplantarum* L-ZS9 through regulating the expression of multiple genes. Front Microbiol 9:2628. 10.3389/fmicb.2018.02628 PMC6240686 30483223

[CR34] Liu Y, Wu L, Han J, Dong P, Luo X, Zhang Y, Zhu L (2021) Inhibition of biofilm formation and related gene expression of *Listeria monocytogenes* in response to four natural antimicrobial compounds and sodium hypochlorite. Front Microbiol 11:617473. 10.3389/fmicb.2020.617473 PMC7840700 33519777

[CR35] Liu Y, Bu Y, Li J, Liu Y, Liu A, Gong P, Liu T, Zhang L, Wang S, Yi H (2022) Inhibition activity of plantaricin <span class="small-caps">Q7</span> produced by *Lactobacillus plantarum*<span class="small-caps">Q7</span> against *Listeria monocytogenes* and its biofilm. Fermentation 8:75. 10.3390/fermentation8020075

[CR36] Liu HY, Prentice EL, Webber MA (2024) Mechanisms of antimicrobial resistance in biofilms. npj Antimicrob Resist. 10.1038/s44259-024-00046-3 PMC11445061 39364333

[CR37] Livak KJ, Schmittgen TD (2001) Analysis of relative gene expression data using real-time quantitative <span class="small-caps">PCR</span> and the 2^−ΔΔCT^ method. Methods 25:402–408. 10.1006/meth.2001.1262 11846609

[CR38] Marini E, Magi G, Ferretti G, Bacchetti T, Giuliani A, Pugnaloni A, Rippo MR, Facinelli B (2018) Attenuation of *Listeria monocytogenes* virulence by *Cannabis sativa* L. essential oil. Front Cell Infect Microbiol 8:293. 10.3389/fcimb.2018.00293 PMC6113369 30186775

[CR39] Mattila M, Lindström M, Somervuo P, Markkula A, Korkeala H (2011) Role of <span class="small-caps">flhA</span> and <span class="small-caps">motA</span> in growth of *Listeria monocytogenes* at low temperatures. Int J Food Microbiol 148:177–183. 10.1016/j.ijfoodmicro.2011.05.022 21683466

[CR40] Mazaheri T, Cervantes-Huamán BRH, Bermúdez-Capdevila M, Ripolles-Avila C, Rodríguez-Jerez JJ (2021) *Listeria monocytogenes* biofilms in the food industry: is the current hygiene program sufficient to combat the persistence of the pathogen? Microorganisms 9:181. 10.3390/microorganisms9010181 PMC7830665 33467747

[CR41] Meireles MCA, Gomes FJ, Silva RC et al (2016) Isolation of *Listeria monocytogenes* in milk and dairy products marketed in the city of Cuiabá, Mato Grosso. Cienc Rural 46:699–704. 10.1590/0103-8478cr20141739

[CR42] Miao X, Liu HH, Zheng Y, Guo D, Shi C, Xu YF, Xia X (2019) Inhibitory effect of thymoquinone on biofilm formation and virulence attributes of *Listeria monocytogenes* ATCC 19115 crucial for human infection. Front Cell Infect Microbiol 9:304. 10.3389/fcimb.2019.00304 PMC6718631 31508379

[CR43] Palomino MM, Allievi MC, Martin JF, Waehner PM, Prado Acosta M, Sanchez Rivas C, Ruzal SM (2015) Draft genome sequence of the probiotic strain *Lactobacillus acidophilus* ATCC 4356. Genome Announc 3:e01421-14. 10.1128/genomeA.01421-14 PMC4299901 25593259

[CR44] Patel K, Panchal R, Sakariya B, Gevariya M, Raiyani R, Soni R, Goswami D (2025) Combatting antibiotic resistance by exploring the promise of quorum quenching in targeting bacterial virulence. Microbe 6:100224. 10.1016/j.microb.2024.100224

[CR45] Pieta L, Escudero FLG, Jacobus AP, Cheiran KP, Gross J, Moya MLE et al (2017) Comparative transcriptomic analysis of *Listeria monocytogenes* reveals upregulation of stress genes and downregulation of virulence genes in response to essential oil extracted from *Baccharis psiadioides*. Ann Microbiol 67:479–490. 10.1007/s13213-017-1277-z

[CR46] Prinzi A, Rohde RE (2023) The role of bacterial biofilms in antimicrobial resistance. American Society for Microbiology. Available at: https://asm.org/articles/2023/march/the-role-of-bacterial-biofilms-in-antimicrobial-re

[CR47] Ribeiro AC, Almeida FAD, Medeiros MM, Miranda BR, Pinto UM, Alves VF (2023) *Listeria monocytogenes*: an inconvenient hurdle for the dairy industry. Dairy 4(2):316–344. 10.3390/dairy4020022

[CR48] Riedel CU, Monk IR, Casey PG, Waidmann MS, Gahan CGM, Hill C (2009) AgrD-dependent quorum sensing affects biofilm formation, invasion, virulence and global gene expression profiles in *Listeria monocytogenes*. Mol Microbiol 71:1177–1189. 10.1111/j.1365-2958.2008.06589.x 19154329

[CR49] Rieu A, Weidmann S, Garmyn D, Piveteau P, Guzzo J (2007) Agr system of *Listeria monocytogenes* EGD-e: role in adherence and differential expression pattern. Appl Environ Microbiol 73:6125–6133. 10.1128/AEM.00608-07 PMC2075002 17675424

[CR50] Rocourt J, Schrettenbrunner A, Seeliger HPR (1983b) Différenciation biochimique des groupes génomiques de *Listeria monocytogenes* (sensu lato). Ann Microbiol (Inst Pasteur) 134A:65–71. 10.1016/0769-2609(83)90104-7 6405670

[CR51] Rocourt J, Schrettenbrunner A, Seeliger HPR 1983. Differénciation biochimique des groupes génomiques de Listeria monocytogenes (sensu lato). Ann Microbiol (Inst Pasteur) 134 A: 65–71. 6405670

[CR52] Rotta IS, Perini HF, Rezende SDC, Sabino YNV, da Silva MV, de Almeida FA, Naves EAA, Pinto UM, Machado ABF, Paiva AD (2025a) Bactericidal, anti-biofilm, and stress-response modulatory effects of *Lacticaseibacillus rhamnosus* ATCC 9595 cell-free supernatant against *Listeria monocytogenes*. Foods 14:4163. 10.3390/foods14234163 PMC12692526 41376100

[CR53] Rotta IS, Rezende SDC, Perini HF, da Silva MV, de Almeida FA, Pinto UM, Machado ABF, Paiva AD (2025b) Exploring the potential of Weissella paramesenteroides UFTM 2.6.1 in disrupting quorum sensing and attenuating virulence in Listeria monocytogenes. Front Microbiol 16:1601203. 10.3389/fmicb.2025.1601203 PMC12213706 40606152

[CR54] Salman MK, Abuqwider J, Mauriello G (2023) Anti-quorum sensing activity of probiotics: the mechanism and role in food and gut health. Microorganisms 11:793. 10.3390/microorganisms11030793 PMC10056907 36985366

[CR55] Sarikhani M, Kermanshahi RK, Ghadam P, Gharavi S (2018) The role of probiotic *Lactobacillus acidophilus* ATCC 4356 bacteriocin on effect of HBsu on planktonic cells and biofilm formation of *Bacillus subtilis*. Int J Biol Macromol 115:762–766. 10.1016/j.ijbiomac.2018.03.087 29567501

[CR56] Sela S, Frank S, Belausov E, Pinto R (2006) A mutation in the luxS gene influences *Listeria monocytogenes* biofilm formation. Appl Environ Microbiol 72:2644–2650. 10.1128/AEM.00048-06 PMC1538747 16885324

[CR57] Shi W, Zhang Q, Li H, Du D, Ma X, Wang J, Jiang J, Liu C, Kou L, Ren J (2024) Biofilm formation, motility, and virulence of *Listeria monocytogenes* are reduced by deletion of the gene lmo0159, a novel Listerial LPXTG surface protein. Microorganisms 12:1354. 10.3390/microorganisms12071354 PMC11278909 39065121

[CR58] Surette MG, Miller MB, Bassler BL (1999) Quorum sensing in *Escherichia coli*, *Salmonella typhimurium*, and *Vibrio harveyi*: a new family of genes responsible for autoinducer production. Proc Natl Acad Sci U S A 96:1639–1644. 10.1073/pnas.96.4.1639 PMC15544 9990077

[CR59] Taga ME, Xavier KB (2011) Methods for analysis of bacterial autoinducer-2 production. Curr Protoc Microbiol Chapter 1:Unit1C.1. 10.1002/9780471729259.mc01c01s23 22045583

[CR60] Thorat SR, Rawool DB, Sonkusale PM, Warke SR, Choudhari SP, Kurkure NV (2019) Virulence profiling of *Listeria monocytogenes* isolated from different sources. Int J Curr Microbiol App Sci 8:2337–2350. 10.20546/ijcmas.2019.805.233

[CR61] Todhanakasem T, Young GM (2008) Loss of flagellum-based motility by *Listeria monocytogenes* results in formation of hyperbiofilms. J Bacteriol 190:6030–6034. 10.1128/JB.00155-08 PMC2519545 18586947

[CR62] Upadhyay A, Upadhyaya I, Kollanoor-Johny A, Venkitanarayanan K (2013) Antibiofilm effect of plant-derived antimicrobials on *Listeria monocytogenes*. Food Microbiol 36:79–89. 10.1016/j.fm.2013.04.010 23764223

[CR63] Vatanyoopaisarn S, Nazli A, Dodd CER, Rees CED, Waites WM (2000) Effect of flagella on initial attachment of *Listeria monocytogenes* to stainless steel. Appl Environ Microbiol 66:860–863. 10.1128/AEM.66.2.860-863.2000 PMC91911 10653766

[CR64] Vilas Boas D, Castro J, Araújo D, Nóbrega FL, Keevil CW, Azevedo NF, Vieira MJ, Almeida C (2024) The role of flagella and flagella-based motility in biofilm formation by *Salmonella Enteritidis* and *Escherichia coli*. Microorganisms 12:232. 10.3390/microorganisms12020232 PMC10893291 38399635

[CR65] Wadhwa N, Berg HC (2021) Bacterial motility: machinery and mechanisms. Nat Rev Microbiol 19:779–793. 10.1038/s41579-021-00626-4 34548639

[CR66] Wang A, Teng Y, Jin T, Zhu W, Jia J, Xia X (2025) Inhibitory effects of the cell-free supernatants of *Faecalibacterium prausnitzii on* biofilm formation of *Listeria monocytogenes*. Food Res Int 213:116588. 10.1016/j.foodres.2025.116588 40436568

[CR67] World Organization for Animal Health (WOAH) (2021) *Listeria monocytogenes*. In: Manual of diagnostic tests and vaccines for terrestrial animals, Chap. 3.10.5. WOAH, Paris.

[CR68] Yu T et al (2022) Andrographolide inhibits biofilm and virulence in *Listeria monocytogenes* as a quorum-sensing inhibitor. Molecules 27:3234. 10.3390/molecules27103234 PMC9145827 35630711

